# Accurate Identification of Native Asian Honey Bee Populations in Jilong (Xizang, China) by Population Genomics and Deep Learning

**DOI:** 10.3390/insects16080788

**Published:** 2025-07-31

**Authors:** Zhiyu Liu, Yongqiang Xu, Wei Sun, Bing Yang, Tenzin Nyima, Zhuoma Pubu, Xin Zhou, Wa Da, Shiqi Luo

**Affiliations:** 1 State Key Laboratory of Agricultural and Forestry Biosecurity, MOA Key Lab of Pest Monitoring and Green Management, College of Plant Protection, China Agriculture University, Beijing 100193, China; zliu90693@gmail.com (Z.L.); b20233191069@cau.edu.cn (W.S.); yangbingheart@163.com (B.Y.); xinzhoucaddis@icloud.com (X.Z.); 2 Jilong Valley Biodiversity Observation and Research Station, Institute of Plateau Biology of Xizang Autonomous Region, Lhasa 858700, China; sws_xyq@sti.xizang.gov.cn (Y.X.); danzeng220227@163.com (T.N.); sws_pz@sti.xizang.gov.cn (Z.P.)

**Keywords:** population genetics, *Apis cerana*, lineage identification, population structure

## Abstract

The Jilong Valley, situated in Rikaze, Xizang, China, is characterized by its complex topography and variable climatic conditions, providing a suitable habitat for *Apis cerana* Fabricius, 1793. To facilitate the conservation of germplasm resources and maintain genetic diversity, it is imperative to elucidate the population structure and lineage differentiation of *A. cerana* within this ecologically distinct region. In this study, we collected *A. cerana* specimens from 12 geographically disparate locations across various altitudinal gradients within the Jilong Valley, and also integrated publicly available sequencing data of *A. cerana* from various regions across mainland Asia. In total, our analysis encompassed sequencing data from 296 individuals. Population structure analyses based on SNP data revealed that *A. cerana* in Jilong represents a genetically distinct population that differs markedly from other regional *A. cerana* populations in terms of genetic lineage, although its subspecies identity remains to be confirmed. Through screening based on F_ST_ values, we identified SNP loci that contribute significantly to distinguishing between Jilong and non-Jilong *A. cerana*. Using these loci, the convolutional neural network model TraceNet was trained, which demonstrated specific recognition capabilities for Jilong versus non-Jilong *A. cerana*. This further confirmed the universality and efficiency of TraceNet in identifying honey bee lineages. These findings contribute valuable insights for the identification and conservation of *A. cerana* germplasm resources in specific geographical regions.

## Simple Summary

The Jilong Valley in Xizang is the habitat of the Asian honey bee, a crucial pollinator for plants across Asia. These bees face growing threats from climate change and human activities, making it essential to understand and protect their unique genetic makeup. This study investigated the genetic characteristics of honey bees in Jilong Valley to support conservation efforts. We collected bee samples from 12 locations throughout the valley and also used data from other Asian regions. By analyzing next-generation sequencing data, we found that Jilong bees represent a distinct genetic group within Asian honey bee populations that has not been documented in previous studies. We also applied an existing artificial intelligence tool called TraceNet to help accurately identify and distinguish Jilong bees from other geographic populations based on their genetic patterns. These findings provide valuable information for creating targeted conservation strategies to protect these important pollinators.

## 1. Introduction

Bees are essential pollinators, playing a critical role in sustaining ecosystem services and enhancing crop yields. Among the native bee species in Asia, *Apis cerana* Fabricius 1793 (Asian honey bee) is particularly important due to its ability to exploit scattered nectar sources, its strong foraging capacity, and its adaptation to diverse environmental conditions [1]. However, since the early 20th century, the introduction of *Apis mellifera* Linnaeus 1758 (Western honey bee) for commercial purposes has exerted considerable ecological pressure on *A. cerana*. This includes competition for floral resources, disruption of nuptial flights, and the introduction of diseases and pests. In addition, habitat loss, pesticide overuse, and environmental pollution have further contributed to the decline of *A. cerana* populations [2,3,4,5]. With the development of the beekeeping industry, the introduction and hybridization of honey bees may unintentionally affect the genetic integrity of local *A. cerana* populations by causing unintended genetic admixture [6,7]. This raises concerns regarding their genetic integrity and long-term survival.

Recent studies have shown that physical barriers such as straits, rivers, and mountains serve as key drivers of genetic differentiation in the population of *A. cerana*. Chen et al. [8] demonstrated that in 18 *A. cerana* populations across China, there was no significant correlation between genetic and geographic distances, suggesting that the influence of physical barriers on genetic differentiation may be greater than that of geographic proximity. This barrier-driven pattern of differentiation has been confirmed across various geographic features. Studies have shown significant genetic and phenotypic differentiation between island populations and mainland populations, or between island populations themselves. Such differentiation has been observed on Damen Island [9], Hainan Island [10], the Sansha Islands [11], and Ko Samui [12,13]. Similarly, mountainous regions have also restricted gene flow, with different genetic structures due to mountain isolation observed in the Loess Plateau [14], the Pyrenees [15], and the Qinghai–Xizang Plateau [16]. Among these regions, the Qinghai–Xizang Plateau presents a particularly compelling case for studying *A. cerana* population dynamics [16]. Due to low temperatures and limited nectar sources, these bees are restricted to altitudes below 3500 m, forming isolated populations within valleys. This valley-dominated distribution pattern limits gene flow among populations, thereby promoting genetic differentiation while simultaneously increasing extinction risks for these small populations. The extreme environmental conditions and complex topography of the Qinghai–Xizang Plateau make it a region of high conservation priority. Efforts to identify and preserve the genetic resources of *A. cerana* in this area are crucial for safeguarding their evolutionary potential and resilience.

The Jilong Valley (28.25°~29.00° N, 85.10°~85.68° E), located in the southern part of Rikaze City, Xizang Autonomous Region, China, is situated in the southwestern region of the Qinghai–Xizang Plateau and represents the westernmost of the five major Himalayan valleys (Zhangmu, Jilong, Yadong, Chentang, and Gama). The valley extends for 93 km, spanning from Mount Mala’s peak (5770 m) to Resuo Village (1700 m), with an average elevation of approximately 4000 m [17]. Due to its complex geological conditions and substantial elevation gradient, the region serves as a transitional zone between the Indomalayan and Palearctic realms [18]. Warm, moist monsoons from the Indian Ocean create distinct climatic zones along the altitudinal gradient, fostering remarkable biodiversity with clear vertical zonation patterns of species distributions. As the core area of the Himalaya National Nature Reserve, the Jilong Valley provides an ideal habitat for *A. cerana*. The absence of introduced *A. mellifera* populations, along with high floral diversity, makes it an exceptional site for studying endemic *A. cerana* lineages.

Currently, research on *A. cerana* lineages in the Jilong region remains notably limited. Ji et al. [19] included six samples from Jilong in their broader study of *A. cerana* across Asia. Qiu et al. [20] further classified the Jilong population as belonging to the *A. c. cerana* subspecies based on these samples. However, the samples used in these studies were collected from locations with similar altitudes and geographic coordinates, lacking comprehensive sampling to reflect the genetic characteristics of Jilong *A. cerana* populations. Therefore, there is a compelling need to conduct new sampling across altitudinal gradients in this region to better understand the genetic diversity and population structure of *A. cerana* in the Jilong Valley.

In this study, we investigate the phylogenetic patterns of *A. cerana* populations in the Jilong Valley and other regions using genome-wide SNP data, integrating traditional population genomics approaches with novel deep learning methods. Specifically, we aim to (1) determine whether the *A. cerana* lineage in Jilong exhibits genetic differentiation from other regional populations, especially the central population; (2) evaluate the performance of the TraceNet [21] model for distinguishing *A. cerana* populations; and (3) identify characteristic SNP loci that distinguish Jilong populations from surrounding *A. cerana* lineages. Our findings will inform the development of targeted conservation strategies to preserve the genetic diversity of these important pollinators in high-altitude environments.

## 2. Materials and Methods

### 2.1. Samples Collection

**Figure 1:**
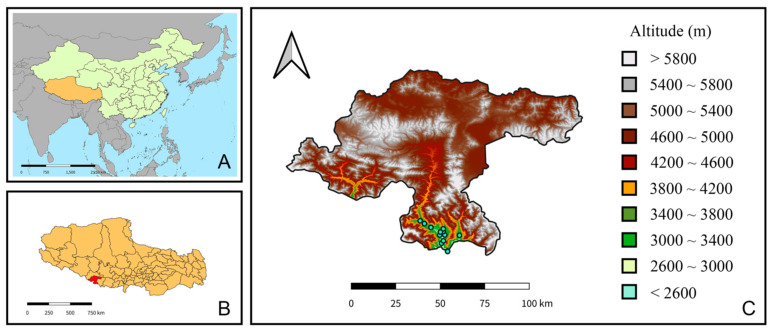
Geographical location and topography of the sampling area. (**A**) The location of Xizang Autonomous Region (orange) within China. (**B**) The location of Jilong County (red) within Xizang. (**C**) The sampling sites and topographic map of Jilong County, showing the altitude gradient (1519~8330 m) with different colors.

All bee samples used for next-generation sequencing were collected from Jilong County, Xizang Autonomous Region, China. The sampling design covered different regions and altitudinal gradients throughout Jilong Valley, comprising a total of 141 worker bees from 12 distinct locations (Table 1 and Figure 1): Jilong–Nepal Port (JNP), Laojiang Village Hot Spring (LJHS), Jilong to Port Shelter No. 1 (JTS), Chongse Village (CSV), Jifu Village (JFV), Xinjiang Village (XJV), Rapeseed Fields Around Jilong Town (RFAG), Sa’le Town (SLT), Maga Village (MGV), Bangxing Community (BXC), Rema Village (RMV), and Naixia Village (NXV). Since most sampling sites had no introduced *A. cerana* populations and wild nests were difficult to locate, foraging bees were collected as extensively as possible using sweep netting. A small number of samples confirmed as introduced *A. cerana* from central populations were obtained from managed hives and served as negative controls during the analysis. All specimens were prepared as dry specimens and deposited at the College of Plant Protection, China Agricultural University, Beijing, China.

### 2.2. DNA Extraction

To ensure representative coverage across all sampling sites, 1 to 3 worker bees were randomly selected from each location, depending on sample availability, resulting in a total of 26 individuals. Thoraxes of the selected individuals were used for DNA extraction. DNA was extracted using the Blood/Cell/Tissue Genomic DNA Extraction Kit (DP304, Catalog No.: 4991108, Tiangen Biotech, Beijing, China), following the manufacturer’s protocol. The DNA samples were stored at −20 °C and sent for sequencing.

### 2.3. Genome Sequencing

De novo whole-genome resequencing was performed by Beijing Novogene Bioinformatics Technology Co., Ltd. (Beijing, China) using the Illumina platform. Genomic DNA was fragmented to approximately 350 bp using a Covaris sonicator (Covaris, Woburn, MA, USA), followed by purification and end repair to generate blunt-ended fragments with 5′ phosphate groups. A dA-tail was added to the 3′ ends of the fragments, which were then ligated to Illumina sequencing adapters using T4 DNA ligase. The adapter-ligated library was purified and size-selected using the Agencourt SPRIselect Nucleic Acid Fragment Selection Kit (Catalog #: 2358413, Beckman Coulter, Brea, CA, USA) to enrich for fragments within the desired size range. The library was further amplified by PCR to obtain sufficient DNA for sequencing. The insert size of the library was evaluated using the Agilent 5400 system (AATI) (Agilent Technologies, Santa Clara, CA, USA), and its concentration was quantified using qPCR to ensure quality. Finally, the prepared library was amplified by bridge PCR, and paired-end 150 bp (PE150) sequencing was conducted on the Illumina platform.

### 2.4. Public Data

**Figure 2:**
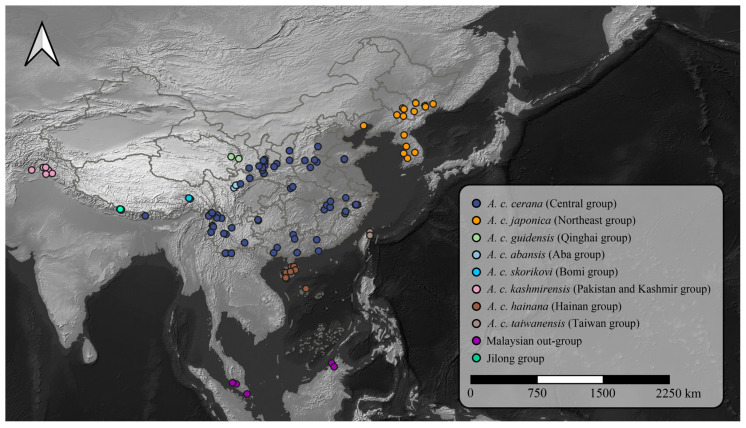
Geographic distribution of *A. cerana* sampling sites across Asia. The map shows the locations of 299 *A. cerana* samples, including 26 newly sequenced individuals from the Jilong Valley and 273 publicly available genome samples from Ji et al. (2020) [19] and Qiu et al. (2023) [20]. Colored dots represent distinct population groups.

To expand the geographic coverage of our study and confirm the identity of *A. cerana* populations in the Jilong region, we integrated public whole-genome resequencing data from *A. cerana* populations in Jilong and other areas, published by Ji et al. [19] and Qiu et al. [20], comprising 273 samples. These datasets were retrieved from public repositories and processed using the same bioinformatics pipeline as our newly sequenced data to ensure comparability in downstream analyses. Based on the population structure defined by Ji et al. [19], all samples were classified into different groups (Figure 2). Although populations in Jilong are generally categorized within the Central group (subspecies: *A. c. cerana*), we designated them as a separate “Jilong group” in this study to facilitate the identification of genomic signatures unique to this lineage.

### 2.5. Quality Control

Quality control of raw sequence data was performed on a CentOS 7 system using FastQC (version 0.12.1; https://github.com/s-andrews/FastQC, accessed on 21 January 2025) and fastp (version 0.23.1; https://github.com/OpenGene/fastp, accessed on 21 January 2025) [22]. FastQC was used to generate comprehensive quality assessment reports for the raw data, while fastp was employed to filter paired-end reads containing adapters, remove reads with excessive N bases, and eliminate low-quality reads. Specifically, paired-end reads were removed under the following conditions: (1) if either read contained more than 10% N bases; (2) if more than 50% of the bases in either read had low quality scores (Q ≤ 5); or (3) if either read contained adapter sequences.

### 2.6. Mapping and Variant Calling

Minimap (version 0.2-r123; https://github.com/lh3/minimap2, accessed on 21 January 2025) [23] was used to index the *A. cerana* reference genome (Ref ID: AcerK_1.0) and align clean reads to the reference. Samtools (version 1.21; https://github.com/samtools/samtools, accessed on 21 January 2025) [24,25] was then used to convert SAM files to sorted BAM format. For variant detection, bcftools (version 1.21; https://github.com/samtools/bcftools, accessed on 23 January 2025) [24] was employed to call short variants, including 25,932,220 SNPs and 940,948 indels. The initial variant set was subsequently filtered to retain high-quality biallelic SNP sites meeting the following criteria: (1) average depth between 1/3 and 2 times the mean depth of the dataset; (2) quality score > 50; (3) average genotype quality > 20; (4) proportion of missing genotypes < 10%; (5) minor allele count > 5; and (6) minor allele frequency > 0.05. The filtered SNP dataset was compressed with bgzip (version 1.21; https://github.com/samtools/htslib, accessed on 21 January 2025) and indexed using tabix (version 1.21; https://github.com/samtools/tabix, accessed on 21 January 2025) for downstream analyses.

### 2.7. Kinship Analysis and Sample Filtering

Among the 26 *A. cerana* samples from Jilong used in this study, 24 were collected in the field rather than from managed colonies. To ensure that our dataset better represented the diversity of local *A. cerana* populations, only one individual per colony was retained for subsequent analyses. To accomplish this, we employed the make-king-table method in plink (version 1.90b6.21; https://www.cog-genomics.org/plink, accessed on 28 February 2025) [26] and relatedness2 in vcftools (version 0.1.16; https://github.com/vcftools/vcftools, accessed on 21 January 2025) [27] to calculate and cross-validate kinship relationships between pairs of Jilong samples. Pairs of individuals with kinship coefficients greater than or equal to 0.375 (3/8) but less than 0.5 were considered full sisters, while pairs with kinship coefficients greater than or equal to 0.125 (1/8) but less than 0.375 were identified as offspring from the same queen mated with different drones. Consequently, pairs of individuals with kinship coefficients greater than 0.125 were classified as belonging to the same colony, and only one individual from each such pair was retained.

### 2.8. Population Structure and Phylogeny

SNP datasets were further filtered using the indep-pairwise function in plink to mitigate the effects of linkage disequilibrium (LD) on population structure and phylogenetic analyses. We employed a window size of 50 SNPs, a step size of 5 SNPs, and an LD threshold of 0.2. Subsequently, whole-genome identical-by-state (IBS) matrices were calculated for the pruned SNP subset using plink and shell scripts. Phylogenetic trees were constructed using Fastme (version 2.1.6.3; http://www.atgc-montpellier.fr/fastme, accessed on 7 March 2025) [28] with the 1-IBS matrix as input, employing the balanced minimum evolution method. Ancestry inference was performed using admixture (version 1.3.0; https://dalexander.github.io/admixture, accessed on 10 April 2025) [29], with totally 14 independent runs conducted for each value of K ranging from 2 to 15. The VCF files were processed using the cyvcf2 (version 1.0.3; https://github.com/brentp/cyvcf2, accessed on 4 May 2025) [30] package, and dimensionality reduction techniques including Principal Component Analysis (PCA), t-distributed Stochastic Neighbor Embedding (t-SNE), and Uniform Manifold Approximation and Projection (UMAP) were applied to the processed SNP datasets using scikit-learn (version 1.6.1; https://github.com/scikit-learn/scikit-learn, accessed on 21 January 2025) [31] and umap-learn (version 0.5.7; https://github.com/lmcinnes/umap, accessed on 4 May 2025) [32] packages. The dimensionality reduction results were visualized using the seaborn package (version 0.13.2; https://github.com/mwaskom/seaborn, accessed on 4 May 2025) [33].

### 2.9. Lineage Classification

For the specific identification of the Jilong population and to evaluate the accuracy and versatility of deep learning approaches in honey bee lineage detection, we employed the convolutional neural network model TraceNet [21]. This model processes SNP data in FASTA format, using population membership of each sample as training labels. To enhance the model’s identification accuracy, we implemented an F_ST_-based SNP filtering approach [34]. Specifically, we calculated F_ST_ values between the Jilong population and all other populations, then applied appropriate thresholds to retain only SNP sites that significantly contributed to lineage differentiation. These filtered, lineage-specific markers were subsequently converted to FASTA format using vcf2phylip (version 2.9; https://github.com/edgardomortiz/vcf2phylip, accessed on 6 May 2025) and served as the input data for TraceNet model training and validation.

The training process followed the methodology outlined by Yang et al. [21]. The dataset was divided into training (75%) and test sets (25%), ensuring balanced representation of different lineages across these sets. The model was trained using a supervised learning approach with a categorical cross-entropy loss function and Adam optimizer. Early stopping was implemented with a patience of 10 epochs to prevent overfitting, and model performance was evaluated based on accuracy and loss metrics. We also applied data augmentation techniques, including random masking of SNP positions, to enhance the model’s generalization capabilities. The final model was selected based on its performance on the validation set.

All computational analyses were performed on a workstation running Arch Linux (kernel: 6.13.3-arch1-1) equipped with 32GB of RAM, a 1TB WD Blue SN580 SSD, an Intel i7-14700KF (28) @ 5.500GHz CPU, and an NVIDIA GeForce RTX 4080 SUPER GPU.

### 2.10. Functional Enrichment Analysis

To investigate whether SNPs that differentiate honey bee populations from Jilong and non-Jilong regions are located within any annotated genes potentially associated with adaptation, we calculated Weir and Cockerham’s F_ST_ for each SNP between the Jilong population and the combined group of all non-Jilong populations using vcftools (version 0.1.16; https://github.com/vcftools/vcftools, accessed on 21 January 2025) [27]. The top 5% of SNPs with the highest F_ST_ values were extracted using bcftools (version 1.21; https://github.com/samtools/bcftools, accessed on 23 January 2025) [24] for subsequent annotation and enrichment analysis. SNP annotation was performed with snpEff (version 5.2; https://github.com/pcingola/SnpEff, accessed on 15 July 2025) [35], for which a custom database was constructed based on the genome of *A. cerana*. Based on the annotation output, candidate genes were extracted using the cyvcf2 Python package (version 1.0.3; https://github.com/brentp/cyvcf2, accessed on 4 May 2025) [30]. The resulting gene list was then subjected to functional enrichment analysis using the DAVID web tool (version 2025_1; https://davidbioinformatics.nih.gov/, accessed on 16 July 2025) [36].

## 3. Results

### 3.1. Sampling and Genome Sequencing

We performed whole-genome resequencing on 26 samples collected from different altitudinal gradients throughout the Jilong Valley. Three pairs of individuals were identified as half-sisters (with the same mother but different fathers) based on kinship analysis; consequently, we kept 23 samples, with only one individual from each pair retained. Additionally, we obtained whole-genome sequencing data for 273 samples from publicly available datasets published in previous studies. In total, this study incorporated individuals representing 296 distinct colonies (one individual per colony), with geographic distributions spanning mainland China, Hainan, Taiwan, Kashmir, Malaysia, and other regions (Table S1). The average sequencing depth across all data was approximately 19-fold. After initial quality control, a total of 1,278,517 SNPs were identified and selected for subsequent analyses.

### 3.2. Population Structure Analysis Indicates Jilong A. cerana Form a Genetically Distinct Lineage

**Figure 3:**
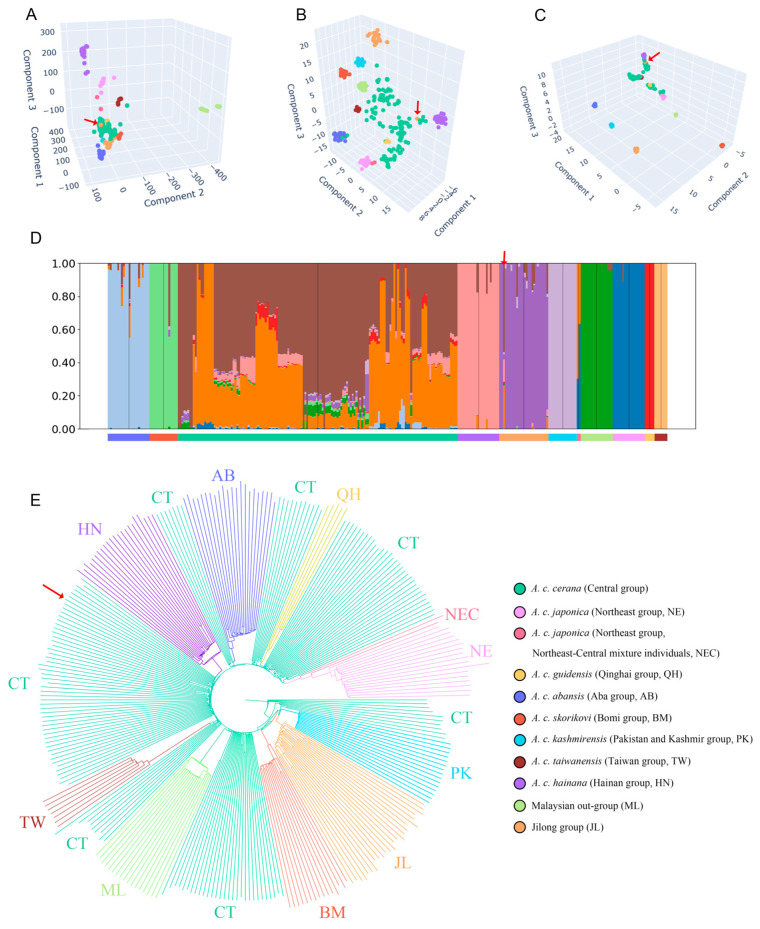
Principal component analysis (PCA) dimensionality reduction results (**A**); t-distributed stochastic neighbor embedding (t-SNE) dimensionality reduction results (**B**); uniform manifold approximation and projection (UMAP) dimensionality reduction results (**C**); population structure assuming 11 ancestral populations (**D**) and minimum-evolution tree (**E**) for 296 *A. cerana* samples. The outlier sample from Jilong is indicated by red arrows.

Population structure analysis based on genome-wide SNP data revealed clear genetic differentiation patterns among *A. cerana* populations. PCA, t-SNE, and UMAP consistently identified one cohesive cluster and one outlier among samples collected from the Jilong region (Figure 3A–C, Files S1–S3). The majority of Jilong samples (n = 22, representing 96% of all Jilong samples) formed a tightly clustered group. In the PCA results, the Jilong, Qinghai, Bomi, and Pakistan–Kashmir clusters were positioned relatively close to the Central group, whereas the remaining populations (Taiwan, Hainan, Northeast, Aba, and Malaysia) were more distantly separated, indicating greater genetic similarity between the former groups and the Central population.

Compared to PCA, the t-SNE analysis revealed a more pronounced clustering of the Jilong population, which contained no samples from other populations, indicating significant genetic differentiation between Jilong bees and other groups. However, due to the non-linear nature of t-SNE, inter-cluster distances lack directly interpretable genetic meaning, limiting conclusions about the exact degree of divergence.

In the UMAP dimensionality reduction results, all populations except the Central group formed distinct clusters. The Central group was primarily divided into four subgroups showing affinity with Hainan, Taiwan, Qinghai, and Northeast populations, respectively. Additionally, a small number of Central samples clustered with other population groups, such as two samples from Yadong, Xizang that grouped with the Jilong cluster.

Across all clustering analyses, one consistent outlier from the Jilong region was identified that clustered with the Central group rather than with the main Jilong population. Upon verification, this sample was confirmed as originating from a managed colony, representing an introduced specimen from Central group rather than a native Jilong bee.

The clustering results were also supported by admixture analysis. When K values ranged from 7 to 11, the cross-validation (CV) error of the admixture results remained relatively low (0.47218~0.48171) (Figure S1). Across all K values, Jilong honey bees consistently exhibited a predominantly single ancestral lineage. At K = 7 or 8, the Pakistan–Kashmir population also showed substantial admixture with the Jilong ancestral component (Figure S1). When K = 11, the Central group primarily displayed a mixture of two ancestral lineages, while the remaining populations each exhibited distinct and relatively homogeneous ancestral compositions, highly consistent with the findings of Ji et al. [19] and Qiu et al. [20]. Therefore, although K = 8 corresponded to the lowest CV error value, the ancestral lineage partitioning did not align with historical conclusions for various populations. Consequently, we selected K = 11 as the optimal K value for interpreting the ancestral composition of the Jilong group (Figure 3D). In the admixture results, we also identified one sample from the Jilong region that exhibited an ancestral composition consistent with the Central group rather than with other Jilong bees, similar to the outlier observed in the dimensionality reduction analyses. This further confirms that this sample represents an introduced bee of the Central group. Several Jilong samples also displayed minor ancestral components from the Central group, indicating some degree of historical gene flow between these two populations.

The phylogenetic results were highly consistent with previous studies (Figure 3E). When the Malaysian population from the Sundaland was used as an outgroup for the minimum-evolution tree, the Aba, Bomi, Hainan, and other populations all appeared to be derived from the Central group on the tree, with each population forming tight clusters that were interspersed and nested within the Central group. The Jilong population showed a notably distinct phylogenetic position, forming a sister group relationship with the Pakistan–Kashmir population, a relationship which was not previously observed among other populations. This indicates that the Jilong population shares a close phylogenetic relationship with the Pakistan–Kashmir group. Additionally, one Jilong honey bee sample that showed proximity to the Central group in both dimensionality reduction and admixture analyses remained affiliated with the Central group in the phylogenetic tree.

### 3.3. F_ST_-Filtered SNPs Enable Accurate Identification and Reveal Functional Divergence in Jilong Honey Bees

Following the thresholds provided by Yang et al. [21], we used five minimum F_ST_ thresholds of 0.5, 0.6, 0.7, 0.75, and 0.8 to screen for SNPs that contributed most significantly to distinguishing the Jilong population from all other populations (when the threshold exceeded 0.8, the number of retained SNPs became extremely low). Notably, in this analysis, the introduced honey bee from the Jilong region identified in the population structure analysis was classified into the “non-Jilong” group. These thresholds resulted in the retention of 4703, 1568, 376, 152, and 56 SNPs, respectively. Subsequently, these filtered SNPs were used for model training with a batch size of 32, a learning rate of 0.001, and training for 150 epochs. The classification results demonstrated that the TraceNet model achieved 100% accuracy in distinguishing Jilong honey bees from non-Jilong honey bees under all threshold conditions. This result validates the conclusion drawn by Yang et al. [21], who stated that F_ST_ filtering based on thresholds helps with accurate lineage identification. Even when populations not included in previous studies were incorporated, the TraceNet model maintained a high level of classification accuracy, demonstrating its broad applicability. This robust performance suggests that the model can be effectively extended to identify and conserve *A. cerana* genetic resources across other geographic regions as well.

**Figure 4:**
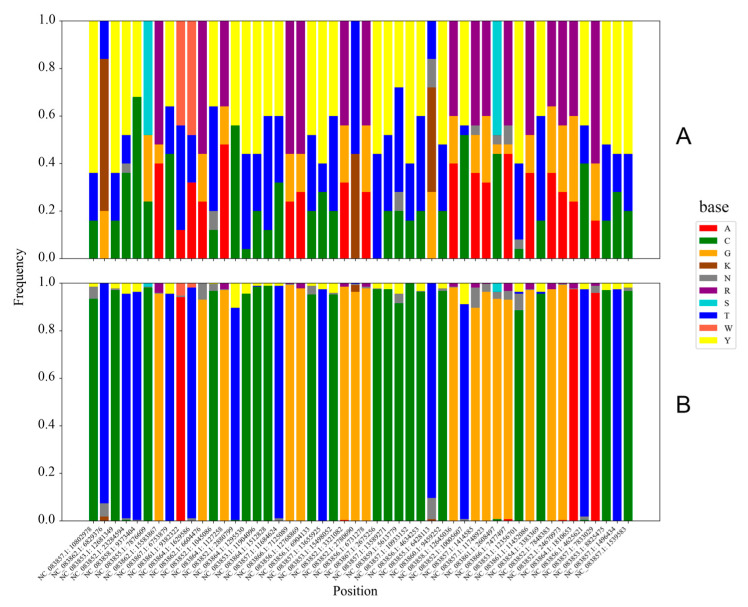
Base composition of the top 50 SNPs with the highest F_ST_ values in Jilong (**A**) and non-Jilong (**B**) samples. Different colors represent different bases.

To determine which SNP loci contributed most significantly to distinguishing between Jilong and non-Jilong honey bees, we extracted the top 50 SNP loci with the highest F_ST_ values, ranging from 0.80446 to 0.92303. Comparison of these loci revealed that non-Jilong populations exhibited relatively uniform nucleotide compositions at these positions, while Jilong populations generally displayed complex patterns of homozygous and heterozygous mutations, with numerous degenerate bases (Figure 4). The most common mutation pattern in the Jilong population was the coexistence of three variants at SNP loci where non-Jilong populations showed a single purine/pyrimidine: the same purine/pyrimidine, the alternative purine/pyrimidine, and heterozygous combinations of both purines/pyrimidines. In rare cases, at positions where non-Jilong populations showed a single homozygous genotype (C, G, A, or T), Jilong samples exhibited all three possible genotypic states: both homozygous alternatives and their heterozygous combination.

Functional enrichment analysis was performed on genes annotated from the top 5% of SNPs with the highest F_ST_ (Tables S2–S4). Gene Ontology (GO) enrichment revealed significant overrepresentation in several biological processes and molecular functions, including proteolysis (GO:0006508), olfactory receptor activity (GO:0004984), serine-type endopeptidase activity (GO:0004252), chitin binding (GO:0008061), and odorant binding (GO:0005549). Enriched cellular components included the extracellular region (GO:0005576) and axoneme (GO:0005930). No significant enrichment was detected for KEGG pathways.

### 3.4. Altitudinal and Spatial Distribution Patterns of Ancestral Components Suggest Concurrent Natural Dispersal and Human-Mediated Introduction of Central Populations in Jilong

**Figure 5:**
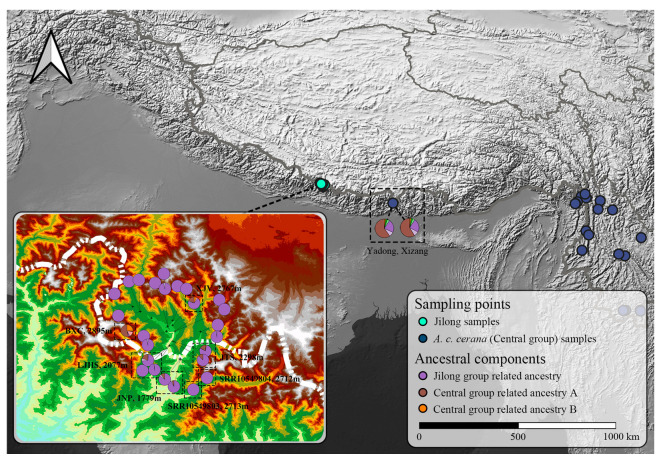
Ancestral component composition and distribution of samples from Jilong and adjacent region (Yadong). Different colors in the pie charts represent distinct ancestral lineages.

To examine whether the Jilong population exhibits mixing patterns with central group at valley entrances similar to other Xizang Plateau valley populations (such as Aba, Bomi, and Qinghai), we sorted the admixture ancestral lineage composition results for the Jilong samples (assuming K = 11 ancestral populations) by altitudinal gradient and conducted visualization (Figure 5 and Figure S2).

The results revealed that the vast majority of Jilong samples exhibited relatively homogeneous ancestral composition (dark purple), with a few samples containing low proportions of Pakistan–Kashmir group ancestry (light purple) and Central group ancestry. Integrating findings from Ji et al. [19] and the present study, the Central group comprised two major ancestral components (brown and orange), with their relative proportions showing a southwest–northeast geographical gradient, where the brown ancestral component predominates in southwestern regions, particularly in Xizang.

Among the Jilong honey bee samples, excluding one introduced foreign specimen, six samples showed admixture with Central group ancestral components. Five of these samples were distributed across the three lowest-elevation sampling sites in the southernmost part of the Jilong Valley: Jilong–Nepal Port (JNP, 1779 m), Laojiang Village Hot Spring (LJHS, 2077 m), and Jilong to Port Shelter No. 1 (JTS, 2298 m). The central ancestral components detected in these samples were consistently brown, matching the dominant component of the nearest central population (Yadong, Xizang). This pattern suggests possible natural dispersal of the Central group from low to high elevations, entering through the valley entrance and naturally hybridizing with the Jilong group.

Notably, the sample from Xinjiang Village (XJV, 2767 m) exhibited a distinctive admixture pattern. Despite being distant from the southern valley entrance of Jilong and situated at relatively high elevation, this sample still displayed substantial proportions of central population admixture. Its orange ancestral component proportion was similar to that of foreign-introduced central group individual among the Jilong samples. Given the close proximity in both horizontal distance and elevation between the specimen, we hypothesize that the admixture in the XJV sample may be associated with human-mediated introduction activities.

## 4. Discussion

Population genetic studies of *A. cerana* across most regions of China indicate that physical barriers, such as mountains and straits, are the primary obstacles limiting gene flow, and thereby leading to population differentiation [8,10,11,14]. Among these, *A. cerana* populations distributed in Xizang Plateau valleys are restricted to valleys below the tree line due to their requirement for tree cavities for nesting, resulting in limited gene flow between valley populations and significant genetic differentiation [16]. Historically, the central subspecies *A. c. cerana*, distributed in the central-eastern regions of mainland China, has repeatedly dispersed into Xizang Plateau valleys and formed subspecies therein [19,20]. Considering the extensive distribution of *A. cerana*, regions not covered by previous studies, such as deep valleys of the Xizang Plateau, central-southern India, or Sri Lanka, may still harbor unrecorded new subspecies [20]. Jilong Valley is located within the currently presumed distribution range of central populations, and its north–south-oriented canyon structure may have facilitated the historical northward migration of central populations to form new subspecies or populations.

In this study, we comprehensively analyzed 296 samples from Jilong Valley and other regions. The results from dimensionality reduction, phylogenetic trees, and admixture analyses consistently demonstrated that native Jilong honey bees possess unique genetic characteristics distinct from other known subspecies. However, compared to Qiu et al. [20], determining the subspecific identity of the Jilong population is beyond the scope of this study due to the lack of morphological data, absence of central population samples from regions adjacent to Jilong, and the inherent difficulties in defining subspecies [37]. Therefore, we define Jilong honey bees merely as a distinctive genetic unit with potential subspeciation tendencies.

Our admixture results differ from those of Ji et al. [19]. Although their previous study also included samples from the Jilong region, those Jilong samples exhibited ancestral compositions consistent with central population, whereas in our study, native Jilong samples displayed ancestral compositions significantly distinct from all other populations. This discrepancy may be attributed to the possibility that when Jilong samples are scarce, algorithms may fail to detect sufficient variation to distinguish the Jilong population from the Central population [29,38]. Similar situations may occur when sampling other high-altitude valley populations. Therefore, adequate sample sizes are crucial for population genetic studies of honey bees in high-altitude valleys, and ancestral inference results based on small samples should be interpreted with caution.

In this study, dimensionality reduction, phylogenetic trees, and admixture analyses all identified an outlier among the Jilong samples. Phylogenetic analysis revealed that this sample was closely related to the Central population from Guangxi, likely representing a foreign-introduced honey bee from Guangxi. Historically, cross-regional introduction of honey bees has been a common practice [10], which can result in varying degrees of lineage admixture. Therefore, when conducting population genetic studies of *A. cerana*, it is essential to consider the possibility of foreign introductions within sampling regions.

Based on these comprehensive results, we can draw preliminary conclusions: The Jilong honey bee represents a population with a relatively distinct lineage composition that has not been clearly documented previously, showing closer relationships to both the Central group and the Pakistan–Kashmir group. However, the specific subspecies identity of this population remains uncertain. Given the available information, it can be considered a unique population or one exhibiting a trend toward subspecies formation. Apart from the native indigenous honey bees in the Jilong region, the introduced honey bees belong to the Central group and share close genetic affinities with the populations from Guangxi Province within this group.

Accurate identification of honey bee lineages is crucial for conservation efforts. Although traditional methods such as phylogenetic tree construction and principal component analysis (PCA) have been widely applied in population genetics [21,39], both approaches have limitations when processing large-scale genomic datasets. Phylogenetic trees require frequent reconstruction whenever new lineages are introduced, making the process computationally expensive and sensitive to sample composition. Meanwhile, PCA only captures directions of maximum variance while ignoring variations in other directions, potentially leading to information loss [40,41]. Recent advances in machine learning and deep learning provide powerful alternatives [42,43,44,45]. For example, in this study, compared to phylogenetic trees and admixture analyses, two unsupervised learning methods, t-SNE and UMAP, demonstrated relatively high efficiency and rapid detection capabilities. The convolutional neural network model TraceNet achieved 100% accuracy in distinguishing Jilong from non-Jilong honey bees when using F_ST_-filtered SNP data as input. TraceNet’s robust performance across different F_ST_ thresholds indicates its broad applicability in honey bee conservation efforts. This method is particularly valuable for rapid field identification of honey bee populations and tracking cross-regional migration of specific lineages.

Gene Ontology enrichment analysis suggest that the genetic divergence of the Jilong population is linked to adaptations in sensory perception, protein metabolism, and potentially structural elements crucial for motility or environmental interaction. Collectively, these enriched functions imply that local ecological pressures, such as dietary variations or specific environmental stimuli within the valley ecosystem, likely played a significant role in shaping the genetic profile of Jilong honey bees.

Ji et al. [19] proposed the formation mechanism of honey bee subspecies in Xizang Plateau valleys. Initially, the Central population dispersed into valleys, and when the Central population was reduced during glacial periods, valleys served as refugia for inner honey bees. The diverse environmental conditions in different valleys promoted differentiation of marginal subspecies, while relatively narrow valley entrances limited gene flow with central subspecies. When central subspecies subsequently re-expanded into valleys, secondary contact and hybridization with marginal subspecies would occur at valley entrances. In this study, ancestral lineage compositions from admixture analysis suggest that a similar pattern may have occurred in Jilong Valley.

The three sampling sites located closest to the valley opening and at the lowest elevations (JNP, LJHS, JTS) all exhibited varying degrees of admixture with the Central populations. Qiu et al. [20] also utilized two samples from near JNP (Figure 5), but these samples did not exhibit admixture with the Central population because they were situated at elevations considerably higher than the JNP site. Additionally, the sample from site XJV, despite not being located near the valley entrance or at low elevation, still displayed substantial proportions of admixture with central population. Given that XJV and the foreign-introduced sample site BXC are relatively close in both horizontal distance and elevation, and their Central population lineage compositions are highly concordant, we hypothesize that the admixture at XJV resulted from foreign-introduced hybridization, while the southern samples resulted from natural dispersal of the Central population.

This study has several unresolved issues. Although 23 representative individuals from Jilong were included to ensure broad geographic coverage, the relatively small sample size may limit the resolution of fine-scale genetic patterns. Future research incorporating larger sample sizes would help validate and refine these findings. Compared to the subspecies determination of the Aba, Bomi, and Qinghai populations, the close-range vicinity of Jilong, particularly the valley entrance region, lacks *A. c. cerana* samples, which creates difficulties for further confirmation of the Jilong population’s identity—specifically, whether it constitutes a true subspecies in the strict sense. Additionally, while Qiu et al. [20] hypothesized that *A. c. cerana* are distributed along the southern Xizang Plateau-northern India line, the actual breadth of *A. c. cerana* distribution range remains unclear. Furthermore, the relationship between *A. cerana* populations distributed in central-southern India and those in mainland China is not well understood. It remains uncertain whether expanding the sample scope, particularly by incorporating more Indian samples, would provide different insights into the identity determination and population history interpretation of Jilong honey bees.

## 5. Conclusions

This study utilized genomic data to analyze *A. cerana* populations distributed in Jilong Valley, investigating the identity of this population, its identification methods, and hybridization patterns with other populations. Native Jilong *A. cerana* represents a population with genetic characteristics distinct from known subspecies, though whether it constitutes an independent subspecific identity remains to be confirmed. The convolutional neural network model TraceNet, when using F_ST_-filtered SNP data, can effectively distinguish Jilong from non-Jilong *A. cerana* populations and can be effectively extended to lineage identification of *A. cerana* in other regions. Foreign-introduced honey bees in the Jilong region may have caused some degree of admixture with native populations. The results of this study provide insights for research on the evolution of *A. cerana* in Xizang Plateau valleys and for conservation efforts.

## Figures and Tables

**Table 1:** Sample information for all *A. cerana* specimens from Jilong.

Sampling Sites	Latitude	Longitude	Altitude (m)	Number of Individuals
Jilong–Nepal Port	28.2767	85.3803	1779	31
Laojiang Village Hot Spring	28.3235	85.3448	2077	22
Jilong to Port Shelter No. 1	28.3380	85.3560	2298	8
Chongse Village	28.3713	85.3637	2611	13
Jifu Village	28.3710	85.3349	2672	2
Xinjiang Village	28.3911	85.3546	2767	1
Rapeseed Fields Around Jilong Town	28.3884	85.3387	2854	5
Sa’le Town	28.3693	85.4481	2928	26
Maga Village	28.4370	85.2468	3068	10
Rema Village	28.4530	85.2220	3149	4
Naixia Village	28.4071	85.3554	3300	15
Bangxing Community	28.4149	85.2844	2895	4

## Data Availability

The newly sequencing data used in this project have been submitted to the Genome Sequence Archive (GSA) database of China National Center for Bioinformation linked to CRA027137.

## Supplementary Materials

The following supporting information can be downloaded at https://www.mdpi.com/article/10.3390/insects16080788/s1, Table S1: All sample information used in this study; Tables S2–S4: Gene Ontology (GO) enrichment results for Biological Process (BP), Cellular Component (CC), and Molecular Function (MF), respectively; Files S1–S3: Complete dimensionality reduction results (HTML format) for Figure 3A–C; Figure S1: Population structure analysis using admixture showing ancestry composition for K = 7 to K = 11; Figure S2: Spatial distribution of ancestry composition across sampling sites in the Jilong region based on K = 11 ancestries.
