# Supplementary figures and images for: Accurate Identification of Native Asian Honey Bee Populations in Jilong (Xizang, China) by Population Genomics and Deep Learning
Source: Insects. 2025 Jul 31;16(8):788. doi: 10.3390/insects16080788 (PMC12386197; doi:10.3390/insects16080788)

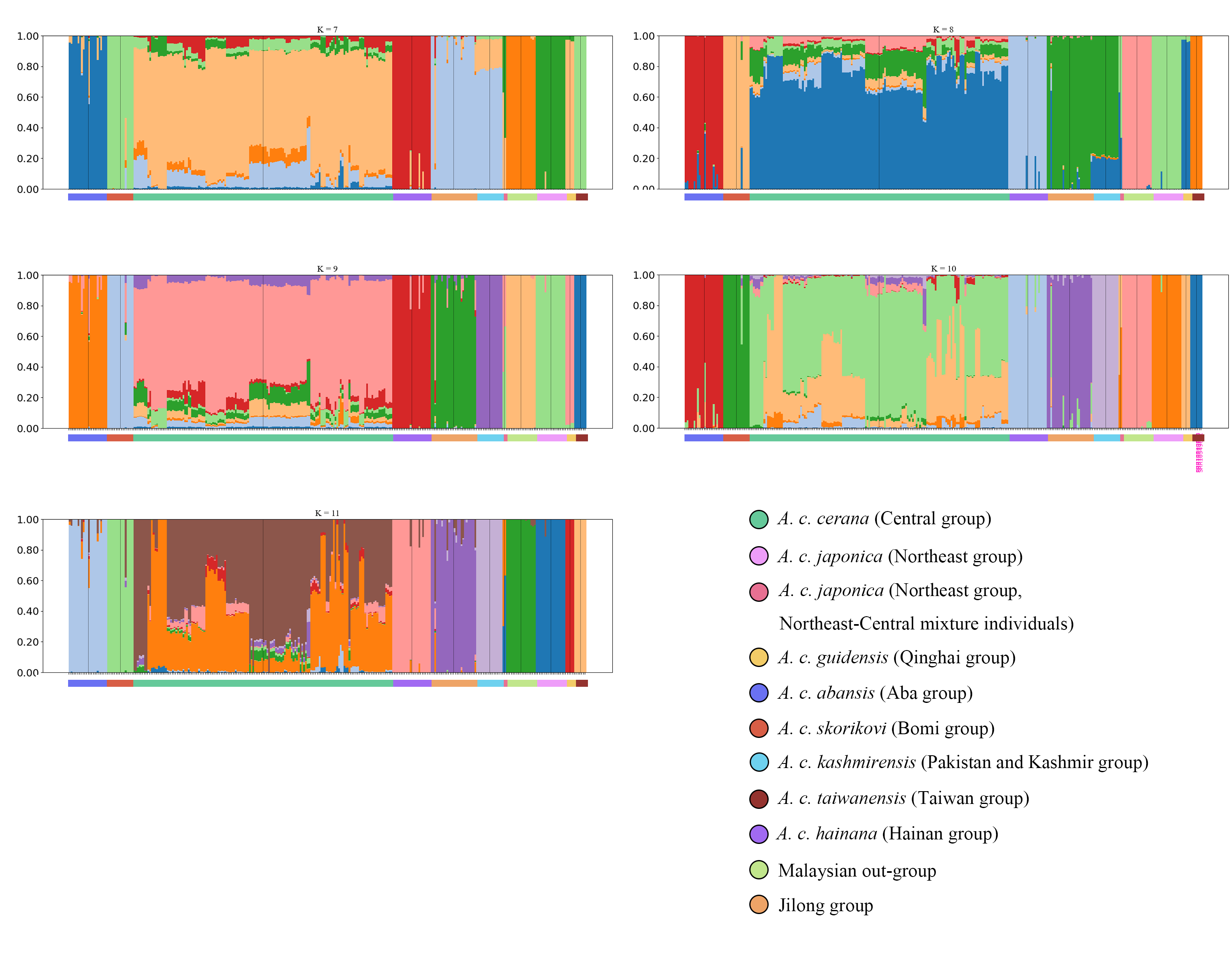

Supplement: Supplementary file 1 [file insects-16-00788-s001.zip › Figure_S1.png]

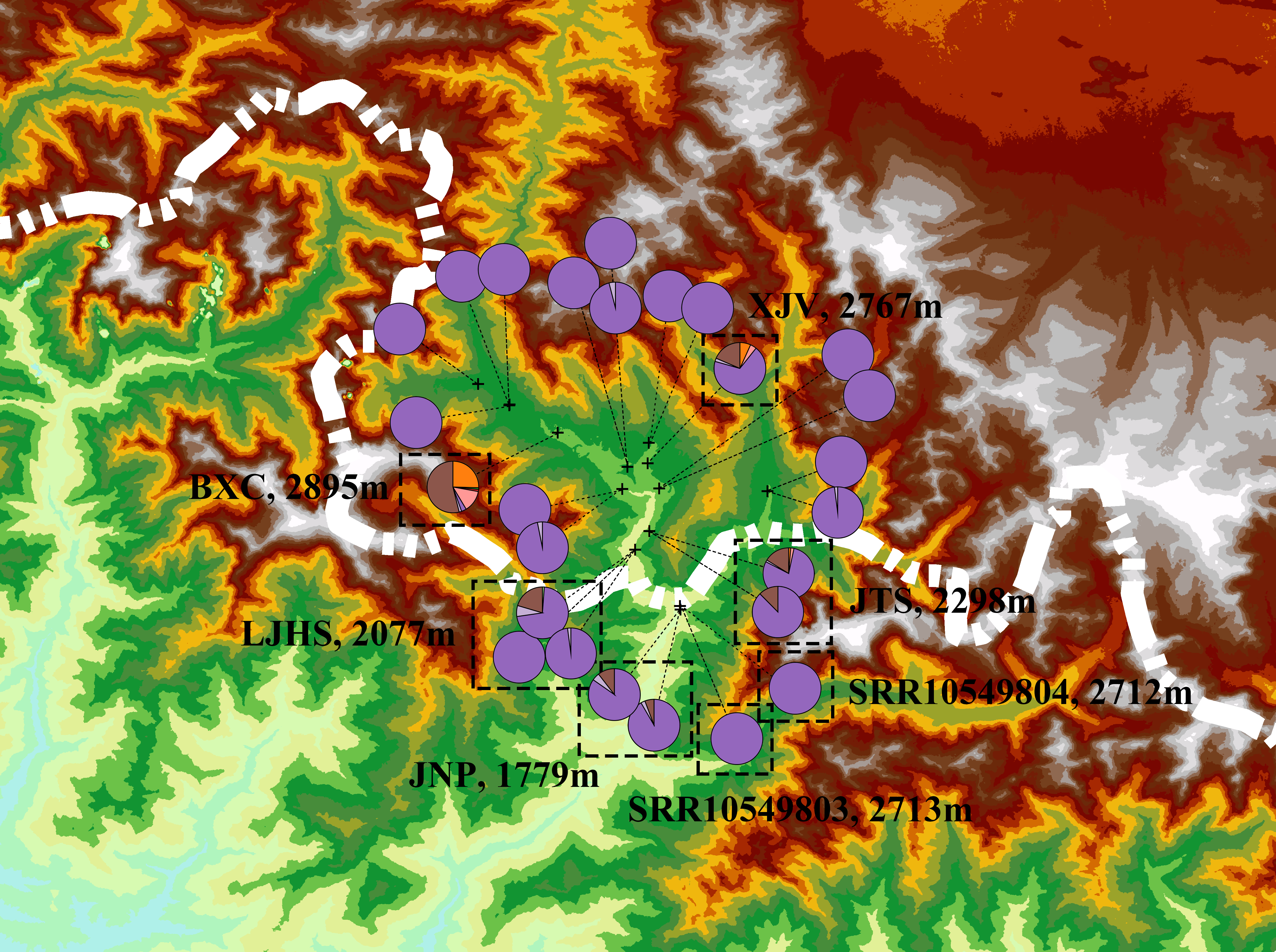

Supplement: Supplementary file 1 [file insects-16-00788-s001.zip › Figure_S2.png]
